# Effectiveness of a community program for older adults with type 2 diabetes and multimorbidity: a pragmatic randomized controlled trial

**DOI:** 10.1186/s12877-020-01557-0

**Published:** 2020-05-13

**Authors:** John J. Miklavcic, Kimberly D. Fraser, Jenny Ploeg, Maureen Markle-Reid, Kathryn Fisher, Amiram Gafni, Lauren E. Griffith, Sandra Hirst, Cheryl A. Sadowski, Lehana Thabane, Jean A. C. Triscott, Ross Upshur

**Affiliations:** 1 Schmid College of Science and Technology, Chapman University, Orange, California 92866 USA; 2 School of Pharmacy, Chapman University, Irvine, California 92618 USA; 3 Faculty of Nursing, University of Alberta, Edmonton, Alberta T6G2R3 Canada; 4 School of Nursing, and Scientific Director, Aging, Community and Health Research Unit, School of Nursing McMaster University, 1280 Main Street West, Hamilton, ON L8S 4K1 Canada; 5 Aging, Community and Health Research Unit, School of Nursing, McMaster University, Hamilton, Canada; 6 McMaster Institute for Research on Aging/Collaborative for Health and Aging (OSSU SPOR Research Centre), Associate Member, Health, Evidence and Impact, McMaster University, 1280 Main Street West, Hamilton, ON L8S 4K1, HSC 3N25B Canada; 7 Aging, Community and Health Research Unit, School of Nursing, McMaster University, 1280 Main Street West, Hamilton, ON L8S 4K1 Canada; 8 Department of Health Research Methods, Evidence, and Impact; and Centre for Health Economics and Policy Analysis, McMaster University, Hamilton, Ontario L8S 4K1 Canada; 9 Department of Health Research Methods, Evidence, and Impact, McMaster University, 1280 Main Street West, Hamilton, ON L8S 4K1 Canada; 10 Faculty of Nursing, University of Calgary, Calgary, Alberta T2N 1N4 Canada; 11 Faculty of Pharmacy and Pharmaceutical Sciences, University of Alberta, 3-171 Edmonton Clinic Health Academy, Edmonton, Alberta T6G 1C9 Canada; 12 Department of Health Research Methods, Evidence, and Impact, McMaster University, Hamilton, Ontario L8S 4K1 Canada; 13 Care of the Elderly Division, Glenrose Rehabilitation Hospital, Rm 1244 10230-111 Avenue, Edmonton, Alberta T5G 0B7 Canada; 14 Department of Family Medicine, Faculty of Medicine & Dentistry, University of Alberta, Edmonton, Canada; 15 Division of Clinical Public Health, Dalla Lana School of Public Health, University of Toronto, Room 678 155 College Street, Toronto, Ontario M5T 3M7 Canada

**Keywords:** Type 2 diabetes mellitus, Comorbidity, Older adults, Self-management, Aging, Community-based program

## Abstract

**Background:**

Type II diabetes mellitus (T2DM) affects upwards of 25% of Canadian older adults and is associated with high comorbidity and burden. Studies show that lifestyle factors and self-management are associated with improved health outcomes, but many studies lack rigour or exclude older adults, particularly those with multimorbidity. More evidence is needed on the effectiveness of community-based self-management programs in older adults with T2DM and multimorbidity. The study purpose is to evaluate the effect of a community-based intervention versus usual care on physical functioning, mental health, depressive symptoms, anxiety, self-efficacy, self-management, and healthcare costs in older adults with T2DM and 2 or more comorbidities.

**Methods:**

Community-living older adults with T2DM and two or more chronic conditions were recruited from three Primary Care Networks (PCNs) in Alberta, Canada. Participants were randomly allocated to the intervention or control group in this pragmatic randomized controlled trial comparing the intervention to usual care. The intervention involved up to three in-home visits, a monthly group wellness program, monthly case conferencing, and care coordination. The primary outcome was physical functioning. Secondary outcomes included mental functioning, anxiety, depressive symptoms, self-efficacy, self-management, and the cost of healthcare service use. Intention-to-treat analysis was performed using ANCOVA modeling.

**Results:**

Of 132 enrolled participants (70-Intervention, 62-Control), 42% were 75 years or older, 55% were female, and over 75% had at least six chronic conditions (in addition to T2DM). No significant group differences were seen for the baseline to six-month change in physical functioning (mean difference: -0.74; 95% CI: − 3.22, 1.74; *p*-value: 0.56), mental functioning (mean difference: 1.24; 95% CI: − 1.12, 3.60; *p*-value: 0.30), or other secondary outcomes..

**Conclusion:**

No significant group differences were seen for the primary outcome, physical functioning (PCS). Program implementation, baseline differences between study arms and chronic disease management services that are part of usual care may have contributed to the modest study results. Fruitful areas for future research include capturing clinical outcome measures and exploring the impact of varying the type and intensity of key intervention components such as exercise and diet.

**Trial registration:**

NCT02158741 Date of registration: June 9, 2014.

## Background

The World Health Organization reports that the number of people with diabetes worldwide was 422 million in 2014 [1]. Approximately 2.3 million Canadians aged 12 years and older reported a diagnosis of diabetes in 2017 [2]. The prevalence of diabetes continues to grow, with the 10 year-risk (2012 to 2022) of developing diabetes in Canadians aged 20 years or older estimated at 9.98% (2.16 million new cases) and associated total costs of diabetes (excluding informal care) estimated at $15.36 billion dollars [3]. It is estimated that in 2018, 17.9% of Canadians aged 65 years and over (1,094,600) had diabetes [4]. Type 2 diabetes mellitus (T2DM) constitutes approximately 90% of all diabetes cases [5] and the management of T2DM is particularly challenging in older adults with multimorbidity. A study in Ontario showed that more than 90% of older adults with diabetes (> 65 years) have at least one comorbid condition and almost 50% have 5 or more comorbid conditions [6]. The number of physician visits, hospitalizations, and associated healthcare costs increases in older adults in proportion to the number of comorbid conditions [6]. There is a need for novel and effective strategies to improve management of T2DM and MCC within the context of limited healthcare services and resources.

Self-management interventions similar to those used in diabetes prevention programs such as the Finnish Diabetes Prevention Study (FDPS) [7, 8] and the U.S. Diabetes Prevention Program (DPP) [9, 10] are recommended for people with T2DM. These programs are multi-faceted and include components such as motivational interviewing, problem solving therapy, patient education and lifestyle modifications. The lifestyle modifications targeted by these programs are appropriate for both diabetes prevention and treatment and have demonstrated effectiveness and long-term sustainability. A recent integrative review of 70 studies summarizing the results of testing 8 types of interventions for T2DM across 17 countries reported mixed findings, but noted that many studies supported small to modest improvements in physiologic, behavioural, and psychological outcomes [11]. This review also noted that self-management programs typically involve a range of professionals and non-professionals working together to provide team-based care. The literature has referred to these programs as integrated care interventions, with the associated systematic reviews reporting mixed results similar to those described above [12].

There is increasing interest in community-based programs, particularly those targeting chronic disease management, because of the resource intensity and resulting high costs of clinic-based programs like the DPP and FDPS, yet the current evidence in support of these programs is weak. A systematic review of studies evaluating the translation of the DPP into primary care, community and work settings reported variable rates of adoption, implementation and sustainability, and recommended better integration of the programs within existing organizational infrastructures (e.g., YMCA) to address these issues [13]. A 2016 Cochrane review of community-based interventions for multimorbidity (many including T2DM) reported no improvements in self-management behaviours, healthcare service use or clinical outcomes and only a small benefit to mental health for programs that targeted specific risk factors or functional limitations [14]. Reliability has been a key concern with existing studies of community-based programs, since many studies use single-group designs or small samples [15]. The effectiveness of these programs is also uncertain in vulnerable groups such as older adults with multimorbidity because they continue to be underrepresented in diabetes research [11, 16, 17]. Further, there has been little cost analysis in previous research [11]. The difficulties in reaching, recruiting and retaining this medically complex population have been reported by others [18].

In summary, the effectiveness of self-management programs for older adults with T2DM and multimorbidity is currently uncertain. More research is needed using objective measures of self-management behaviours, pragmatic designs to provide realistic estimates of treatment effects, and robust cost analyses [11]. This paper presents the results of a trial that addresses these current gaps in the diabetes literature. It reports the results for the Alberta arm of a multisite pragmatic RCT examining whether a 6-month self-management program for older adults with T2DM and multimorbidity was more effective in improving health outcomes than usual care [19]. Results from the pilot study and Ontario arm of this trial are reported elsewhere [20, 21].

### Primary and secondary objectives

Primary Objective: To compare the effect of a 6-month community-based intervention versus usual care on physical functioning in older adults with type 2 diabetes mellitus and 2 or more comorbidities.

Secondary Objective: To compare the effect of a 6-month community-based intervention versus usual care on mental health, depressive symptoms, anxiety, self-efficacy, self-management, and healthcare costs in older adults with type 2 diabetes mellitus and 2 or more comorbidities.

## Methods

A multi-site, pragmatic, randomized controlled trial was conducted in Alberta, Canada. In accordance with the Pragmatic Explanatory Continuum Indicator Summary-2 tool [22], this intervention used a pragmatic approach in the recruitment of participants representative of the population presenting in clinical practice and the flexible delivery of the intervention by clinicians. The effectiveness of a 6-month self-management program for community-dwelling older adults with T2DM and MCC was assessed in comparison to a control group receiving usual care. Details of the study design and outcomes are reported in the published study protocol [19].

### Participants & recruitment

Participants were recruited from three Primary Care Networks (PCNs) in the Edmonton, Alberta, Canada census metropolitan area. PCNs are primary care clinics comprised of family physicians, nurses, nurse practitioners, dietitians, pharmacists, social workers and mental health professionals. The three PCNs were selected for this study because they were similar to one another in terms of primary care services for people with diabetes, provider skills, and older adults served. Study recruitment was conducted from September, 2015 to October, 2016. Clients who had been enrolled at the PCN within the past 24 months were screened (*n* = 725) by employees of the PCNs for potential inclusion and were eligible to participate if they were 65 years or older, living in the community, able to speak English, diagnosed with T2DM by a physician, self-reported at least 2 other chronic conditions (see Supplemental File - Table 1 for full list of conditions), not planning to leave the community during the 6-month study period, if they passed a cognitive screening assessment (achieved at least 5 correct responses on the Short Portable Mental Status Questionnaire [23], and if they were referred to or participated in a chronic disease management program at the participating PCNs within the previous 24 months. Clients were excluded if they were living in a long-term care facility or if someone in the same household was also enrolled in the study.

### Randomization

**Fig. 1:**
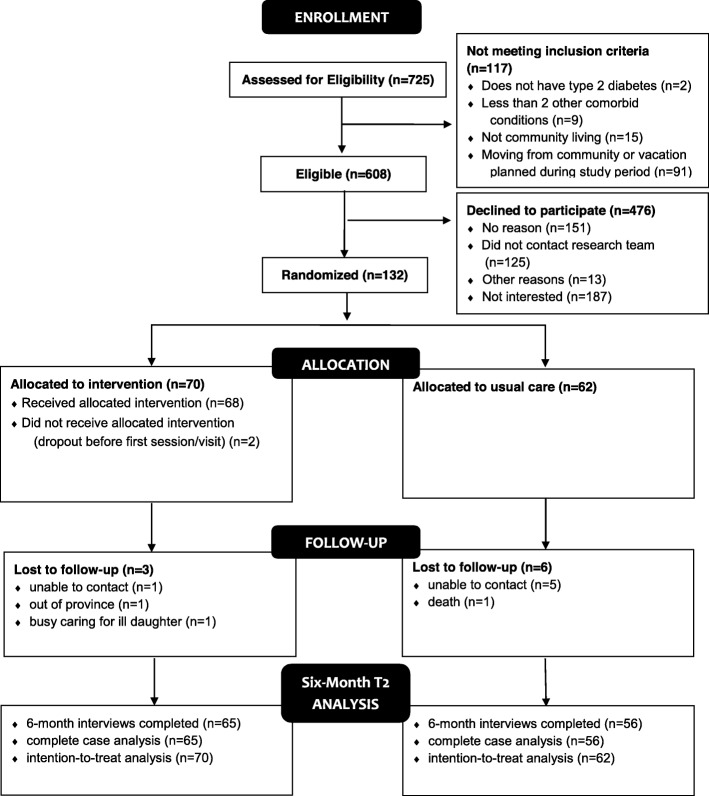
Study Flow Diagram

Of the 608 eligible clients, 132 (22%) provided their written consent and were enrolled in the study. The study flow is outlined in Fig. 1. Reasons for exclusion from study participation and for declining study participation are reported in Fig. 1. Within each PCN, participants were assigned to either the intervention or the usual care group using permuted block randomization (random block size sequences of 2, 4 and 6) administered by a centralized web-based service (RedCap) independent of the research term that allocated clients at each site to the 2 groups in accordance with the sequence and using a 1:1 ratio.

### Intervention

The Aging, Community and Health Research Unit-Community Partnership Program (ACHRU-CPP) was delivered by an interprofessional team consisting of a Registered Nurse (RN), Registered Dietitian (RD), and Program Coordinator (PC). RNs and RDs were employees of the PCNs. PCs were employees of community partner organizations where the monthly group sessions were hosted. Community partner organizations were selected based on pre-existing relationships with respective PCNs. Below we provide a brief summary of the intervention, and refer those seeking further information to the prior publications [20, 21].

The six-month intervention was grounded in Bandura’s Social Cognitive Theory [24], which recognizes the central role of self-efficacy in achieving (self-management) behavior change. The intervention was co-developed with older adults, family caregivers, and community service providers (e.g., family physicians, RNs) and designed using the guidelines for developing complex interventions [25], best practice guidelines for diabetes [26], and empirical evidence on managing diabetes in older adults, The intervention included several interacting components: 1) up to three in-home visits by the RN, RD or both; 2) six monthly group sessions at the community site; 3) monthly intervention team case conferences; and 4) care coordination led by the RN who worked collaboratively with the other interventionists and PCN providers (e.g., family physicians) and linked the client to other relevant health or social services. Family caregivers were invited to attend the in-home visits and monthly group sessions, to recognize their key role in supporting the older adult participants. The monthly group sessions included education offered by the RN or RD, exercise led by the PC, a light meal, and informal peer support (i.e., participants in the group session supported one another by sharing experiences, challenges and strategies for managing diabetes within the context of multimorbidity). Monthly case conferences enhanced team collaboration and communication by providing the provider team with regular opportunities to share observations about participants strengths, challenges and goals related to managing diabetes and other chronic conditions; identify needs related to other community-based services; and prepare for upcoming group sessions. The intervention was tailored to patient needs and preferences and the local context. For example, patients could decline any number of home visits or group sessions, and all participants continued to have access to the programs and services normally offered by the PCN. At one of the PCNs, in order to be consistent with usual care, the intervention team also included a Pharmacist and Kinesiologist. In this PCN, the kinesiologist took on a similar role to the PC in the other sites.

#### Intervention training and fidelity

The following evidence-based strategies [27] were used to monitor the intervention and enhance implementation fidelity:

1. *Training/Educational Workshops*: The Research Coordinator led a training session for the providers (i.e., RNs, RDs, PCs) before implementation of the intervention at the study sites. Training at the sites occurred over two-days. Each session was supported with role-appropriate training manuals. The training focused on intervention components and acceptable ways to tailor them, guidance on interprofessional collaboration (including working with family physicians and other care providers), education and role-playing to develop skills in motivational interviewing, promoting self-management, best practices for the prevention and management of multimorbidity, and caregiver assessment and support strategies. Examples of acceptable ways to tailor the intervention included the number and approximate timing of in-home visits and goals/activities/timeline outlined in the client care plan,
2. *Monthly Implementation Meetings:* The Principal Investigator (PI) and Research Coordinator conducted monthly meetings with the providers to discuss the progress of the study, provide feedback and education, and discuss challenges and potential solutions related to implementation of the intervention. Through this strategy, the research team supported the providers who were implementing the intervention and gave them protected time to reflect on the intervention, share lessons learned, and support one another’s learning.
3. *Reminders:* The PI and Research Coordinator provided regular updates on the study to the providers, including successes and areas for improvement related to the intervention.
4. *Audit and feedback:* The providers were asked to keep logs of intervention-specific activities that were carried out (i.e., home visits, case conferences). At one-month intervals, the PI and Research Coordinator conducted audits of the study-related documentation to assess fidelity by reviewing the extent to which the providers adhered to delivering the components of the intervention. A comprehensive audit and feedback system has been shown to be effective when combined with education, outreach visits, or reminders [28].

### Outcomes & measures

Details regarding outcome measures are described in the study protocol [19]. The primary study outcome was the Physical Component Summary (PCS) score from the 12-item Medical Outcomes Study Short Form-12v1 Health Survey (SF-12) [29]. Secondary outcomes included the Mental Component Summary (MCS) score of the SF-12 [29], depressive symptoms assessed by the Centre for Epidemiological Studies Depression Scale (CESD-10) [30], anxiety assessed by the Generalized Anxiety Disorder (GAD-7) scale [31], self-efficacy assessed by the Self-Efficacy for Managing Chronic Disease scale [32], and self-management activities assessed by the Summary of Diabetes Self-Care Activities (SDSCA) scale [33]. Healthcare use was measured using the Health and Services Utilization Inventory (HSSUI) [34–36]. The cost analysis applied unit costs to the service volumes reported in the HSSUI [37] and assumed a societal perspective in order to inform the broad allocation of resources in the public interest [38]. Outcome measures were assessed at baseline and at the 6-months follow-up.

Guidelines are available for judging clinical significance for only some study measures. SF-12 developers suggest minimally important difference (MID) of 3 for interpreting group mean summary score differences (PCS, MCS) and warn against comparing subdomain scores over time. The CESD-10, GAD-7, Self-Efficacy for Managing Chronic Disease, and SDSCA do not have established MIDs.

### Blinding

In order to reduce bias, the statistician and research assistants that collected the assessment data were blinded. It was not feasible to blind participants or providers.

### Sample size

Sample size was calculated based on the primary outcome measure, the PCS from the SF-12. The target enrollment was 160 clients to account for an expected 20% attrition and to ensure 80% power with medium effect size (0.50), and (two-sided) alpha equal to 0.05.

### Statistical analysis

The reporting of this trial follows the CONSORT guideline (www.consort-statement.org). We applied intention-to-treat principle to analyze the data. Data are presented as means and standard deviations for continuous variables and as numbers and percentages for categorical variables. Analysis of covariance (ANCOVA) was used to test the differences in study outcomes between the intervention and usual care groups at 6 months. Separate ANCOVA models were run for each outcome where the 6-month outcome was the dependent variable, the group (intervention, usual care) was the independent variable, and the baseline outcome value was the covariate. Confidence intervals (95%) are reported for mean differences. Multiple imputation (*n* = 132) was employed using the procedure described by He [39]. A complete case analysis (*n* = 121), performed using only clients with complete outcome data, was conducted as a sensitivity analysis and the results are provided in Supplemental File (Table 2).

Subgroup analyses were proposed for the case where overall trial results achieved statistical significance, with the purpose being to examine the consistency of the treatment effect across subsets of participants. Subgroup analyses would be supplemental to main trial findings and restricted to consideration of the following five baseline factors selected a priori: age, number of chronic conditions, sex, self-efficacy, and diabetes duration. We hypothesized that the intervention may be less effective in participants that are older, female, or have more chronic conditions, lower self-efficacy, or have been living longer with diabetes. No subgroup analyses were proposed for the case where overall trial results did not achieve statistical significance, because these are considered inappropriate by the scientific community [40–42].

The cost of health service use was compared between the intervention and usual care groups. Clients reported health service use at baseline for the six-month period preceding study enrolment and immediately following completion of the six-month intervention. The program cost was estimated and added to total health service cost for the intervention group and compared to the usual care group. Program costs included the payment to providers to deliver the intervention (e.g., time spent in in-home visits, group sessions, team conferences, travel). Mann-Whitney U-test was employed to evaluate differences in median costs between the two groups due to the positive skew of the data. All statistical analyses were performed using SAS Version 9.4 (SAS Institute, Inc., Cary NC).

### Ethics approval and consent to participate

This study was conducted in accordance with the Tri-Council Policy Statement, *Ethical Conduct for Research Involving Humans* [43]. Institutional ethics approval was obtained from the Health Panel of the Health Research Ethics Board at the University of Alberta (Pro00054028) and renewed yearly as required. Operational approval to conduct the research study was obtained from each PCN. Written informed consent was obtained from all participants.

## Results

### Study site characteristics

Table 1 provides information related to the characteristics of the study sites. Sites 1 and 2 served suburban/rural geographies. Half of the intervention teams at these two sites, were Certified Diabetes Educators, and both sites had PCs from a community partner organization to run group sessions. Site 3 served an urban/metropolitan geography, the intervention team included a Pharmacist and Kinesiologist in addition to RN and RD; the interventionists at this site did not have the Certified Diabetes Educator credentials, and the kinesiologist was replaced 3 months into the program.

**Table 1:** Study Site Characteristics

Characteristic	Site 1	Site 2	Site 3
Geographic Density	Suburban/rural	Suburban/rural	Urban/metropolitan
Clinics within PCN	13	11	35
Intervention Team	1 RN, 1 RD	2 RN, 2 RD	1 RN, 1 RD, 1 Pharmacist, 1 Kinesiologist
Program Coordinator (#)	1	1	0
Intervention Team turnover	none	none	Kinesiologist replaced 3 months into the program
Certified Diabetes Educator (#)	1	2	0
Community Partner Organization	yes	yes	no
Participants Enrolled	24	47	61

*PCN* Primary Care Network, *RD* Registered Dietitian, *RN* Registered Nurse

### Baseline characteristics of participants

In total, 22% (132/608) of eligible older adults consented and entered the study (Fig. 1). Of the 132 enrolled participants, 121 completed the 6-month study follow-up (92%, 65 in the intervention and 56 in the control group). Of the 11 participants that did not complete study follow-up at 6 months (< 9% attrition), six participants could not be contacted.

Baseline characteristics of participants are reported in Table 2. For both groups, more than 42% of participants were 75 years and older and more than half were married or living with a partner. Income differences were seen across groups, with one-third of participants in the intervention group reporting an annual income less than $40,000 (CAD) compared to half of the participants in the usual care group. Over 75% of both groups reported having six or more chronic conditions (in addition to T2DM). At least half of participants in both groups were diagnosed with T2DM between 5 and 20 years ago, and approximately 30% were diagnosed with T2DM more than 20 years ago. There were a higher number of females allocated to the intervention group (63%) than the usual care group (45%). Group differences at baseline were also seen for some outcomes, including higher scores for the intervention group for MCS (55.6 versus 52.5) and self-management (37.3 versus 34.2), and fewer depressive symptoms in the intervention group (5.0 versus 6.7). It is notable that the mean group difference for MCS exceeded the MID (3.0), whereas the mean group difference in PCS scores (0.70) was well below the MID. MIDs do not exist for the other outcome measures; however, mean baseline scores were similar across groups.

**Table 2:** Baseline Characteristics of Older Adults with Type 2 Diabetes and Multiple Chronic Conditions (*n* = 132)^a^

**Characteristic**	**Intervention Group (*n*** **= 70)**	**Usual Care Group (*n*** **= 62)**
Female^b^, n (%)	44 (62.9)	28 (45.2)
Age in years, n (%)		
65–69	19 (27.9)	19 (30.6)
70–74	19 (27.9)	17 (27.4)
75+	30 (44.1)	26 (41.9)
Marital Status, n (%)		
Married, living together	42 (60.9)	36 (58.1)
Never Married, Widowed, Divorced, Separated	27 (39.1)	26 (41.9)
Annual Income ($CAD), n (%)		
$0 to $39,999	17 (33.3)	26 (50.0)
$40,000+	34 (66.7)	26 (50.0)
Number of chronic conditions, n (%)		
0–5	13 (18.6)	6 (9.7)
6–10	32 (45.7)	34 (54.8)
11–15	21 (30.0)	19 (30.6)
> 15	4 (5.7)	3 (4.8)
Number of prescription medications, n (%)		
0–3	8 (11.4)	10 (16.1)
> 3	62 (88.6)	52 (83.9)
Time since diabetes diagnosis		
0 to < 5 years	9 (13.6)	13 (21.0)
≥ 5 and < 20 years	37 (56.1)	32 (51.6)
≥ 20 years	20 (30.3)	17 (27.4)
Self-Efficacy^c^, mean (SD)	8.0 (1.6)	7.9 (1.5)
HRQoL - Physical Functioning^d^, mean (SD)	42.4 (10.5)	43.1 (11.7)
HRQoL - Mental Functioning^b,e^, mean & SD	55.6 (7.7)	52.5 (9.3)
Depressive Symptoms^f^, mean (SD)	5.0 (5.2)	6.7 (5.6)
Anxiety^g^, mean (SD)	2.9 (3.9)	2.9 (3.8)
Self-Management^h^, mean (SD)	37.3 (11.7)	34.2 (9.3)

*HRQoL* health-related quality of life, *SD* standard deviation

^a^Differences between the groups were tested using the chi-square test for categorical variables or the t-test for continuous normally-distributed variables

^b^*p* < 0.05, Randomization resulted in allocation of more females to ACHRU-CPP than intervention group

^c^Measured by Self Efficacy for Managing Chronic Disease 6-item Scale, scale range 0–10

^d^Measured by Physical Component Summary Score (PCS) of SF-12 survey, scale range 0–100

^e^Measured by Mental Component Summary Score (MCS) of SF-12 survey, scale range 0–100

^f^Measured by Center for Epidemiologic Studies Depression 10-Item Scale (CES-D-10), scale range 0–30

^g^Measured by Generalized Anxiety Disorder 7-Item Scale (GAD-7), scale range 0–21

^h^Measured by Summary of Diabetes Self-Care Activities Scale (SDSCA), scale range 0–63. SDSCA normally consists of 11 items (2 general diet, 3 special diet, 2 exercise, 2 blood glucose monitoring, 2 ft care). Two blood glucose monitoring items were excluded from the scale score because 45 (34%) of study participants indicated that they did not have a monitoring plan

### Intervention dose

Of the 70 participants in the intervention group, 68 (97%) received at least one home visit and 61 (87%) attended at least one group session. Participants had an average of 1.8 (median = 2) out of a maximum of 3 home visits and attended an average of 3.7 (median = 4) out of a maximum 6 group sessions (data not shown).

### Effects of the intervention

**Fig. 2:**
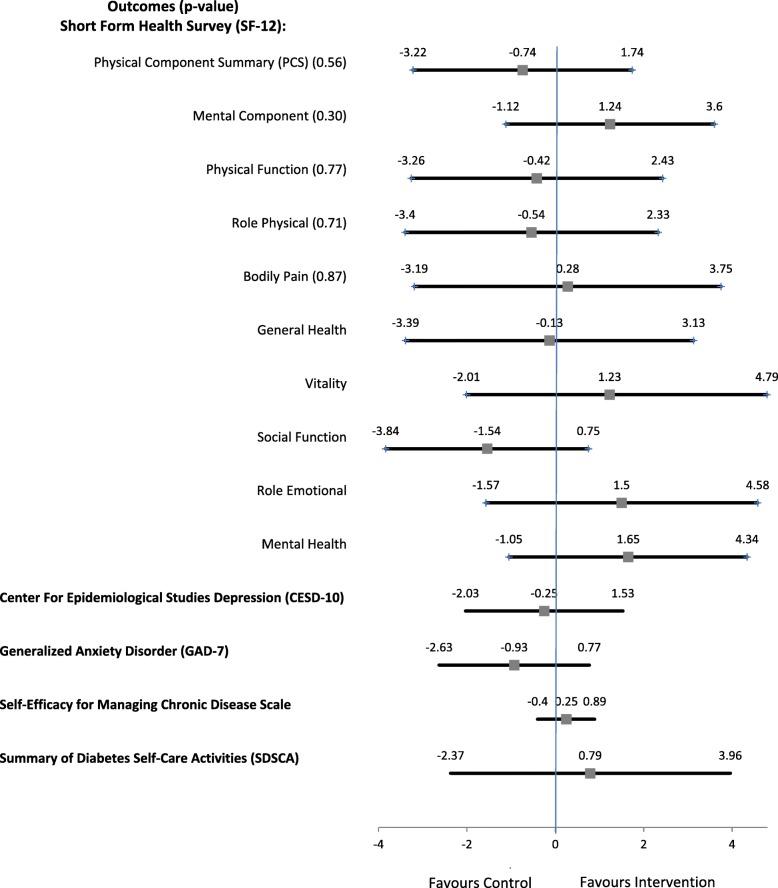
Group Differences in Outcomes (Multiple Imputation, *n* = 132, 5 imputations)

Pooled results from five imputations are presented in Fig. 2. There was no significant difference between the intervention and usual care groups for the primary outcome, PCS (mean difference: -0.74; 95% CI: − 3.22 to 1.74). There were no significant differences between the groups for MCS score (mean difference: 1.24; 95% CI: − 1.12 to 3.60), depressive symptoms (mean difference: -0.25: 95% CI: − 2.03 to 1.53), anxiety (mean difference: -0.93; 95% CI: − 2.63 to 0.77), or self-management (mean difference: 0.79; 95% CI: − 2.37 to 3.96). Complete case analyses were performed as a sensitivity analysis, and the results are presented in Supplemental Table 1. The complete case findings are in agreement with the multiple imputation analysis for the primary and secondary outcomes (see Supplemental Table 1). Subgroup analyses were not done since statistical significance was not achieved (see Statistical Analysis above).

Given the relatively high MCS scores at baseline observed in the intervention group, we conducted an exploratory stratified analysis comparing the mean differences in the groups for different MCS baseline values. This analysis was done to see if improvements in MCS were higher among those with lower baseline MCS scores (defined as ≤50). This was indeed the case – i.e., the mean difference and 95% CI for those with a baseline MCS score of 50 or less compared to those with more than 50 was 2.85 (− 3.42 to 9.12) versus 0.42 (− 2.05 to 2.91). This analysis is supplemental and exploratory, and therefore should be interpreted with caution; however, it suggests that larger variation occurs in participants with lower baseline scores, with the potential for significant differences favouring the intervention (e.g., 9.12 upper CI limit which is more than 3 times the MID).

### Health service use costs

A comparison of health service use costs for the intervention and control groups is presented in Table 3. For all but diabetes care services, the cost analysis showed no significant differences in baseline to 6-month cost change between the two groups. As expected, the significant difference in diabetes care cost change between the groups was due to the inclusion of intervention program costs in the intervention group. However, this significant difference in diabetes care cost change did not result in a group difference in total health service costs (including or excluding acute care costs).

**Table 3:** Health and Social Service Cost Comparison of Intervention and Control Groups (*n* = 121 complete case analysis)

**Service**	**Intervention**		**Usual Care**		**Group Difference**^**a**^	
	**Baseline Median (Q1 to Q3)**	**6 Month Median (Q1 to Q3)**	**Baseline Median (Q1 to Q3)**	**6 Month Median (Q1 to Q3)**	**z stat**	***p*value**
Family Physician Visits	$370.00 ($0.00 to $740.00)	$555.00 ($0.00 to $925.00)	$370.00 ($0.00 to $761.74)	555.00 ($185.00 to $740.00)	0.39	0.70
Physician Specialist	$66.34 ($0.00 to $132.68)	$74.09 ($0.00 to $181.85)	$62.78 ($0.00 to $137.94)	$47.68 ($0.00 to 149.52)	−0.85	0.40
Acute Care^b^	$0.00 ($0.00 to $510.30)	$0.00 ($0.00 to $0.00)	$0.00 ($0.00 to $0.00)	$0.00 ($0.00 to $510.30)	0.84	0.40
Home Care	$0.00 ($0.00 to $0.00)	$0.00 ($0.00 to $0.00)	$0.00 ($0.00 to $0.00)	$0.00 ($0.00 to $0.00)	1.97	0.05
Diabetes Care^c^	$0.00 ($0.00 to $57.43)	$362.20 ($0.00 to $495.74)	$0.00 ($0.00 to $60.00)	$0.00 ($0.00 to $88.48)	−5.57	< 0.001
Diagnostic Tests	$176.19 ($0.00 to $311.07)	$165.88 ($0.00 to 293.46)	$125.05 ($0.00 to $306.92)	$138.77 ($0.00 to $288.19)	−0.22	0.82
Other Health Professionals^d^	$149.94 ($0.00 to $378.52)	$29.30 ($0.00 to $161.25)	$106.71 ($0.00 to $414.43)	$0.00 ($0.00 to $83.60)	−0.26	0.80
Prescription Medications	$870.19 ($65.71 to $1570.69)	$882.54 ($41.56 to $1446.05)	$984.92 ($0.00 to 1594.31)	$857.93 ($58.88 to $1362.68)	−0.68	0.50
Total Costs (including Acute Care)	$2419.49 ($220.35 to $4480.67)	$2528.01 ($678.25 to $3769.45)	$2283.63 ($103.93 to $4002.23)	$2084.60 ($465.32 to $3865.82)	−0.44	0.66
Total Costs (excluding Acute Care)	$2168.27 ($220.35 to $3345.99)	$2442.51 ($678.25 to $3360.00)	$2100.65 ($103.93 to $3006.94)	$1787.83 ($465.32 to $3246.22)	−1.24	0.22

^a^Wilcoxon Rank Sum test used to determine significance of group differences. *P* values are 2-sided

^b^Includes 911 calls, ambulance, emergency department, hospitalization

^c^Includes program costs for the intervention group (training, in-home visits, group sessions, case conferences). Difference between intervention and control group favors control group

^d^Includes provider of private services (e.g., pharmacist, dietitian, social worker, chiropodist, health care aide, physiotherapist, occupational therapist, homemaking service) and alternative therapies (e.g., naturopath, chiropractor, reflexologist)

## Discussion

This was a six-month intervention to test the effects of a community-based program for older adults with T2DM and multimorbidity on physical and mental functioning, self-efficacy, depressive symptoms, anxiety, self-management and service use costs. Although the overall benefits of the intervention were inconclusive for physical and mental functioning, the program shows the potential for significant improvements in mental functioning in participants with lower baseline scores and the program was shown to be cost neutral relative to usual care.

The feasibility, acceptability, and benefits of the program were demonstrated previously in a feasibility study in Ontario [20]. Further, this pragmatic intervention was also conducted in older adults with multimorbidity from Diabetes Education Centres in Ontario, Canada [21]. In Ontario, the intervention improved MCS score, SDSCA, and depressive symptoms [21]. Additionally, certain components of the intervention tested in our studies have been shown to be effective in others. For example, a study with exercise-based intervention showed improvement in body weight, blood pressure and several quality of life measures in older adults with T2DM [44]. This study differed from the present study in that the program was more focused on physical exercise, more intense (three times per week versus monthly) and the intervention lasted much longer (2 years). Another exercise-based study in older adults with T2DM involving walking, resistance training, and flexibility exercises showed an improvement in cognitive function testing after 2 years [45]. Participants in the present intervention in Alberta and Ontario were provided with exercise resources and were encouraged to track their activity on calendars provided to them by the intervention team. Activity monitoring was not conducted in this study, but represents a potential outcome of interest in future trials testing the effectiveness of community-based interventions in older adults with T2DM and MCC.

Despite randomization, mean baseline MCS scores in the intervention group were more than three points higher (MID = 3.0) [46] than the usual care group (Table 3). This may be helpful in understanding the inconclusive findings of this study. Exploratory analysis suggests that the mean difference in MCS score change (favoring the intervention) was higher for those having lower baseline MCS scores (scores ≤50). Further research is needed to replicate and confirm this exploratory finding.

We also explored contextual factors that may have contributed to the inconclusive findings of this trial. It is possible that in the current study, the quality of usual care for persons with diabetes was already good; this would be consistent with a recent study of a lifestyle intervention for persons with T2DM conducted at four PCNs in Alberta [47]. In the current study, there were also a number of changes in the staff and managers of the PCNs, which may have impacted the adoption of the intervention and thus the results; this instability of managers was also noted as an important contextual factor in two quality improvement intervention studies with persons with T2DM conducted with PCNs in Alberta [48]. The background and experience of providers in diabetes care also differed among the sites, which may have impacted the findings.

The intervention arm of the current study included the intervention in addition to usual care. Usual care may have differed slightly across the PCNs, but we are not aware of large differences across the sites. The original protocol for the study included only an RN and RD as the intervention teams. In establishing implementation across study sites, it was deemed that a Pharmacist and Kinesiologist should be included in the usual or standard of care for older adults with T2DM and MCC at one of the PCNs. Accordingly, and in keeping with the principles of the pragmatic trial, a Pharmacist and Kinesiologist were included on the intervention team for this study site (Table 1). As almost half of participants were recruited from this particular study site, higher baseline health measures discussed previously (MCS) or improved T2DM self-management may have reduced the comparative benefits of the intervention. It is also possible that the chronic disease management services offered by PCNs as part of usual care contributed to the lack of statistically significant findings. Further research is needed to better understand the influence of these contextual factors on study outcomes.

**Fig. 3:**
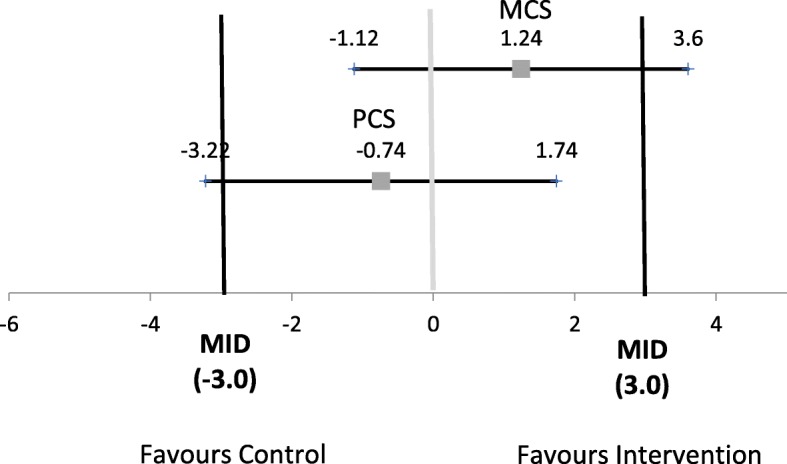
Interpreting 95% Confidence Intervals for PCS and MCS (“Inconclusive” Findings). Note: MID: minimally important difference

A recent systematic review of RCTs reporting non-significant results emphasized the importance of interpreting confidence intervals in order to distinguish “negative” findings from “inconclusive” ones [49]. The authors point out that the *p*-value alone does not allow readers of an RCT to distinguish whether: 1) the treatment does not have a clinically meaningful impact, and 2) the study is unable to rule out a clinically meaningful treatment effect, resulting in “inconclusive” findings. The authors recommend examining confidence limits in relation to the MID (not simply in relation to the 0 or 1 threshold accompanying the effect measure). We can apply this recommendation to our outcome data for which MIDs are available (PCS, MCS). Figure 3 provides a graphic illustration of the interpretation of the MCS and PCS findings for the primary analysis. For MCS, the findings would be considered inconclusive in terms of the superiority of the intervention but rule out the superiority of the control because the upper 95% control limit is above the MID but the lower limit does not extend to the MID favouring the control. For PCS, the findings would be considered inconclusive in terms of the superiority of the usual care group but rule out the superiority of the intervention because the lower limit extends beyond the MID favouring the control but the upper limit does not extend past the MID favouring the intervention [49].

We should also acknowledge the recruitment challenge encountered in this study. Only 22% of eligible participants agreed to participate in the study. This low uptake is disappointing, but is not uncommon and has been cited by other studies of older adults with multimorbidity, such as the 3D trial [50] where 33% of eligible participants agreed to participate and the Guided Care cluster trial [51] where 38% agreed to participate. Recruitment challenges appear to be a significant and realistic barrier to studying vulnerable older adult populations, and effective solutions to the recruitment/retention challenges remain elusive and a priority for future research.

Finally, we acknowledge the role of HbA1C as an important outcome to obtain in future trials testing diabetes interventions. While our study did not have access to HbA1C measures, we recognize it as a key indicator of diabetes and a potential motivator for both physicians and patients. However, including it as an outcome introduces significant interpretive challenges as well, due to the biologic and patient-specific factors common in older adults that can render HbA1C measures misleading/inaccurate (e.g., comorbid conditions, acute illness episodes, hospitalizations). It is therefore critical to apply an individualized patient lens to HbA1C measures and changes over time, when judging the effectiveness of interventions [52, 53].

### Strengths, limitations and areas for future research

This study employed a rigorous pragmatic design. Personnel employed by the site and external to the research team delivered the program. Intervention fidelity was enhanced by multiple approaches, including standardized provider training., and regular meetings were held with the research team. The pragmatic design captured clients from a population typically seen in a clinic setting for chronic disease management. Utility of a RD in the ACHRU-CPP intervention allowed for a holistic approach to dietary counselling that extends beyond calorie restriction to goal setting, general healthy eating, and food preparation. This is particularly important in strategies for older adults as considerations for body mass distribution (lean tissue vs. visceral adiposity) may be more important than targeted weight loss alone [54]. Additionally, the inclusion of a RD in the intervention team is a strength as intensive dietary support is recommended for older adults, men, and individuals whom have been living with T2DM for a long time [55].

Study limitations include the use of self-reported data and the unavailability of clinical outcome data (e.g., HbA1c). However, concerns have been expressed regarding the relevance of clinical outcomes [56], recent work on patient-reported outcomes (PROMs) is motivated by these concerns [see http://www.healthmeasures.net/explore-measurement-systems/promis/intro-to-promis], and PROMs such as the PCS used in this study are increasingly used as primary outcomes in interventions for older adults with multimorbidity [50, 51]. We were unable to obtain the target sample (enrolled 132, needed 160), which could have led to the study’s non-significant findings. With the exception of outcomes from the SF-12 survey (PCS, MCS), MIDs have not been established for the other outcomes included in this study. Thus, distinguishing “negative” effects from “inconclusive” ones by comparing the 95% CI to MIDs could not be done for outcomes other than PCS & MCS, thereby limiting our understanding of the impact of the intervention. This advanced interpretation of CIs in clinical trials is particularly important because it ensures that potentially beneficial interventions are not prematurely abandoned [49]. Interpretation of subscales/subdomains can also be limited. For example, although there was improvement in the Specific Diet subscale score (see Supplemental Table 1, complete case analysis), Toobert and Johnson caution against over-interpretation because this subscale suffers from poor consistency and low internal reliability [33]. The duration of the intervention and the length of follow-up may have been insufficient to see improvements to physical health, and/or it may be that improvements to mental functioning/health are needed before improvements in physical health can be seen. Strong cross-effects between mental and physical health have been reported; however little is known about the potential pathways through which mental health affects physical health and vice versa [57] Subgroup analyses were not performed due to the overall non-significant findings, but as a result we cannot assess the potential impact of differences such as a gender imbalance among the groups or differences across sites. We did not have access to information on important covariates such as physical activity and nutritional intake, which may have influenced the study outcomes.

This study also suggests potential areas to pursue in future research studies. Some studies that have focused on physical exercise have been effective, thus exploring the impact of physical activity components that vary in type or intensity within our intervention may be beneficial. Similar comments apply to other components of our intervention (e.g., diet, social participation), since our intervention is complex making it challenging and potentially inappropriate to isolate the effectiveness of individual elements [25]. Inclusion of clinical outcome measures such as HbA1C could also help in understanding effects, providing careful attention is given to the levels/changes appropriate and safe for individual older adults enrolled in the study.

## Conclusions

This pragmatic trial of an interprofessional, intersectoral self-management intervention for older adults with T2DM and multimorbidity conducted in primary care networks in Alberta demonstrated inconclusive results for physical and mental functioning (PCS and MCS of the SF-12). It is possible that contextual factors may have contributed to these inconclusive results, such as an already good quality of chronic disease management services in PCNs. Further, it is possible that baseline differences between groups may have played a role in the results obtained. Further research on the impact of this intervention and the factors that contribute to the results seen is recommended.

## Supplementary information

**Additional file 1: Supplemental Table 1.** List of Chronic Conditions. **Supplemental Table 2.** Group Differences in Outcomes (Complete Case Analysis).

## Data Availability

The datasets generated and/or analysed during the current study are not publicly available as the participant consent forms did not address open public access to the data and due to limitations of the research ethics approval by the Health Panel of the Health Research Ethics Board (HREB) at the University of Alberta. Data are available upon request from the corresponding author on reasonable request and subject to HREB review.

## Abbreviations

ACHRU-CPP: Aging, Community and Health Research Unit Community Partnership Program
CES-D-10: Centre for Epidemiological Studies Depression scale
GAD-7: Generalized Anxiety Disorder 7-item scale
MCC: Multiple chronic conditions
MCS: Mental component summary
MID: Minimally important difference
PCN: Primary Care Network
PCS: Physical component summary
RCT: Randomized controlled trial
RE-AIM: Reach, Effectiveness, Adoption, Implementation and Maintenance
SDSCA: Summary of Diabetes Self-Care Activities
T2DM: Type 2 diabetes mellitus
