# Supplementary material for: Effectiveness of a community program for older adults with type 2 diabetes and multimorbidity: a pragmatic randomized controlled trial
Source: BMC Geriatr. 2020 May 13;20:174. doi: 10.1186/s12877-020-01557-0 (PMC7218835; doi:10.1186/s12877-020-01557-0)
Supplement: Supplementary file 1 — Additional file 1: Supplemental Table 1. List of Chronic Conditions. Supplemental Table 2. Group Differences in Outcomes (Complete Case Analysis). [file 12877_2020_1557_MOESM1_ESM.docx]

Supplemental Table 1: List of Chronic Conditions

| **Chronic condition** |
| --- |
| **Cardiovascular** |
| Cardiovascular disease (include angina, previous heart attack, atrial fibrillation, lower limbs circulation problems) |
| Stroke (cerebrovascular accident or transient ischemic attack) |
| Hypertension (high blood pressure) |
| Heart failure (including heart valve disease or replacement) |
| Cholesterol problem |
| **Respiratory** |
| Asthma or lung problem like chronic bronchitis, emphysema, or COPD (chronic obstructive pulmonary disorder) |
| Other lung conditions (e.g. pulmonary fibrosis cystic fibrosis) |
| **Mental/Mood Disorders** |
| Depression |
| Anxiety |
| Schizophrenia or bipolar disease |
| **Gastrointestinal** |
| Stomach problem (reflux or peptic ulcer symptoms) |
| Colon problem (chronic inflammatory disease or irritable bowel syndrome) |
| Obesity |
| Constipation |
| Bowel obstruction |
| **Endocrine** |
| Diabetes |
| Thyroid disorder |
| **Liver** |
| Chronic liver disease (including chronic hepatitis or cirrhosis) |
| **Kidney and Urogenital Disorders** |
| Chronic kidney disease or failure |
| Recurrent urinary tract infection |
| Bladder problems (including cystitis, prolapse or repair) |
| Bladder incontinence (does not need to be confirmed by a doctor) |
| Fecal incontinence (does not need to be confirmed by a doctor) |
| Gout |
| Prostate disorders |

Supplemental Table 2: Group Differences in Outcomes (**Complete Case Analysis**)

| **Outcome** | **Intervention (Mean±SD)** | | **Usual Care (Mean±SD)** | | **Group Diff^a^ (95% CI)** | **p-value** |
| --- | --- | --- | --- | --- | --- | --- |
|  | **Baseline** | **6 Months** | **Baseline** | **6 Months** |  |  |
| **HRQoL: SF-12 (n=65 ACHRU-CPP, n=56 usual care)** | | | | | | |
| Physical Function | 42.62±10.37 | 44.23±10.09 | 44.55±11.70 | 45.26±10.40 | 0.87 (-2.33 to 4.07) | 0.59 |
| Role Physical | 44.64±10.15 | 45.16±9.74 | 44.92±10.82 | 45.45±10.91 | 0.01 (-3.41 to 3.42) | 0.99 |
| Bodily Pain | 46.21±11.22 | 46.77±10.87 | 46.62±11.66 | 46.29±11.85 | 0.88 (-3.12 to 4.88) | 0.67 |
| General Health | 49.01±9.39 | 49.83±8.14 | 47.47±10.57 | 49.24±9.86 | -0.95 (-4.26 to 2.36) | 0.57 |
| Vitality | 48.31±9.39 | 51.03±9.71 | 48.01±10.39 | 48.71±11.25 | 2.02 (-1.24 to 5.29) | 0.22 |
| Social Function | 52.52±7.88 | 52.25±7.72 | 51.34±8.90 | 52.77±8.13 | -1.70 (-4.26 to 0.85) | 0.19 |
| Role Emotional | 52.92±6.65 | 52.36±7.00 | 48.76±9.61 | 49.97±11.55 | -1.77 (-4.93 to 1.40) | 0.27 |
| Mental Health | 53.62±8.97 | 55.30±8.67 | 51.61±9.50 | 52.94±7.65 | 0.35 (-3.21 to 3.90) | 0.85 |
| PCS | 42.12±10.44 | 43.17±10.01 | 44.01±11.87 | 44.32±10.11 | 0.74 (-2.19 to 3.68) | 0.62 |
| MCS | 56.04±7.54 | 56.69±8.37 | 52.39±9.53 | 53.88±7.67 | -0.85 (-3.61 to 1.91) | 0.54 |
| **Self Efficacy^b^ (n=62 ACHRU-CPP, n=50 usual care)** | | | | | | |
| Stanford | 8.06±1.60 | 8.29±1.34 | 7.98±1.57 | 7.92±1.87 | 0.31 (-0.27 to 0.88) | 0.3 |
| **Depressive Symptoms: CES-D-10 (n=63 ACHRU-CPP, n=50 usual care)** | | | | | | |
| CES-D-10 | 4.70±5.09 | 4.94±4.46 | 6.90±5.84 | 5.82±4.93 | 1.32 (-0.34 to 2.97) | 0.12 |
| **Anxiety: GAD-7 (n=65 ACHRU-CPP, n=54 usual care)** | | | | | | |
| GAD-7 | 2.89±4.02 | 2.37±3.32 | 3.04±3.98 | 3.24±3.68 | -0.73 (-1.95 to 0.50) | 0.24 |
| **Self Management: SDSCA (9-items)^c^ (n=65 ACHRU-CPP, n=56 usual care)** | | | | | | |
| General Diet | 5.60±1.75 | 5.65±1.67 | 5.37±1.77 | 5.51±1.69 | -0.10 (-0.79 to 0.60) | 0.78 |
| Specific Diet^d^ | 4.53±1.36 | 5.04±1.11 | 4.84±1.73 | 4.61±1.69 | 0.74 (0.12 to 1.36) | 0.02 |
| Exercise | 3.05±2.90 | 2.90±2.15 | 2.88±2.30 | 2.89±2.26 | -0.16 (-1.06 to 0.73) | 0.72 |
| Foot Care | 3.02±2.37 | 3.30±2.34 | 2.54±2.54 | 3.29±2.51 | -0.47 (-1.48 to 0.53) | 0.35 |
| SDSCA | 36.95±11.31 | 38.82±9.71 | 36.09±9.95 | 37.20±11.06 | 0.75 (-3.28 to 4.78) | 0.71 |
|  |  |  |  |  |  |  |
| ^a^ACHRU-CPP intervention mean - usual care mean. Results from ANCOVA adjusted for baseline values | | | | | | |
| ^b^ Measured by Self Efficacy for Managing Chronic Disease 6-item Scale | | | | | | |
| ^c^ Measured by Summary of Diabetes Self-Care Activities Scale. SDSCA normally consists of 11 items (2 general diet, 3 specific diet, 2 exercise, 2 blood glucose monitoring, 2 foot care). Two blood glucose monitoring items were excluded from the scale score because 45 (34%) of study participants indicated that they did not have a monitoring plan. | | | | | | |
| ^d^ Significant interaction exists between covariate and outcome. See interaction plots (supp) for more detail. | | | | | | |
| CES-D-10 = Centre for Epidemiological Studies Depression Scale; GAD-7 = Generalized Anxiety Disorder scale; HRQoL = health-related quality of life; MCS = mental component summary; PCS = physical component summary; SDSCA = summary of diabetes self care activities | | | | | | |
|  |  |  |  |  |  |  |
|  |  |  |  |  |  |  |
